# Functional similarity affects similarity in partner composition in flea-mammal networks

**DOI:** 10.1007/s00436-024-08229-7

**Published:** 2024-05-06

**Authors:** Boris R. Krasnov, Irina S. Khokhlova, M. Fernanda López Berrizbeitia, Sonja Matthee, Juliana P. Sanchez, Luther van der Mescht

**Affiliations:** 1 Mitrani Department of Desert Ecology, Swiss Institute of Dryland Environmental and Energy Research, Jacob Blaustein Institutes for Desert Research, Ben-Gurion University of the Negev, Sede Boqer Campus, 84990 Midreshet Ben-Gurion, Israel; 2 French Associates Institute for Agriculture and Biotechnology of Drylands, Jacob Blaustein Institutes for Desert Research, Ben-Gurion University of the Negev, Sede Boqer Campus, 84990 Midreshet Ben-Gurion, Israel; 3 Programa de Conservación de los Murciélagos de Argentina (PCMA) and Instituto de Investigaciones de Biodiversidad Argentina (PIDBA)-CCT CONICET Noa Sur (Consejo Nacional de Investigaciones Científicas y Técnicas), Facultad de Ciencias Naturales E IML, UNT, and Fundación Miguel Lillo, Miguel Lillo 251, 4000 San Miguel de Tucumán, Argentina; 4 Stellenbosch University, Private Bag X1, Matieland, 7602 South Africa; 5 Centro de Investigaciones y Transferencia del Noroeste de la Provincia deBuenos Aires – CITNOBA (CONICET-UNNOBA), Ruta Provincial 32 Km 3.5, 2700 Pergamino, Argentina; 6 Department of Zoology and Entomology, University of the Free State, 205 Nelson Mandela Dr, Park West, Bloemfontein, 9301 South Africa

**Keywords:** Fleas, Functional dendrogram, Mammals, Mantel test, Traits

## Abstract

*Functional signal* in an interaction network is a phenomenon in which species resembling each other in their traits interact with similar partners. We tested the *functional signal* concept in realm-specific and regional flea-host networks from four biogeographic realms and asked whether the species composition of (a) host spectra and (b) flea assemblages is similar between functionally similar flea and host species, respectively. Analogously to testing for phylogenetic signal, we applied Mantel tests to investigate the correlation between flea or host functional distances calculated from functional dendrograms and dissimilarities in sets of interacting partners. In all realm-specific networks, functionally similar fleas tended to exploit similar hosts often belonging to the same genus, whereas functionally similar hosts tended to harbour similar fleas, again often belonging to the same genus. The strength of realm-specific *functional signals* and the frequency of detecting a significant *functional signal* in the regional networks differed between realms. The frequency of detecting a significant *functional signal* in the regional networks correlated positively with the network size for fleas and with the number of hosts in a network for hosts. A *functional signal* in the regional networks was more frequently found for hosts than for fleas. We discuss the mechanisms behind the *functional signal* in both fleas and their hosts, relate geographic *functional signal* patterns to the historic biogeography of fleas and conclude that *functional signals* in the species composition of host spectra for fleas and of flea assemblages for hosts result from the interplay of evolutionary and ecological processes.

**Supplementary Information:**

The online version contains supplementary material available at 10.1007/s00436-024-08229-7.

## Introduction

The main evolutionary aim of any living organism is to extract resources from its environment and translate these resources into their offspring. Obviously, the successful acquisition of resources requires presence of particular adaptations. For parasitic organisms, including ectoparasitic insects, the resources are provided by their hosts. Moreover, a host provides parasites not only with food resources, but also with a place for living, mating and reproducing. A parasite’s exploitation of a host often causes a loss of fitness in the host, who is thus forced to defend itself against parasitism. The parasite, in turn, develops adaptations not only to extract resources from a host but also to overcome its defence efforts. Ectoparasitic insects possess multiple morphological, physiological, behavioural and ecological adaptations to parasitism (Marshall 1981; Lehane 2005; Krasnov 2008). These adaptations are manifested as a variety of functional traits. For example, chewing lice, parasitic on mammals, have a semicircular head groove that allows them to grasp the hair of the host. However, different host species obviously differ in the diameter of hair-shafts, so the width of a louse’s head groove correlates positively with the diameter of a host’s hair-shaft (Morand et al. 2000). This suggests that successful parasitism requires matching between certain parasite traits and certain host traits in the evolutionary arms race.

Tight links between parasite and host traits (McQuaid and Britton 2013; Mendlová and Šimková 2014; Poulin 2021) can be translated into both parasite and host community structure in the following fashion. The species composition of a host spectrum is expected to be similar in parasites with similar traits, whereas the species composition of a parasite assemblage is expected to be similar in hosts with similar traits. The effects of parasite traits on the diversity of their host spectra or the effects of host traits on the diversity of their parasite assemblages are well known (e.g., Morand and Harvey 2000; Poulin and Morand 2004; Krasnov et al. 2004; Bordes et al. 2011; Mendlová and Šimková, 2014; Dáttilo et al. 2020). On the contrary, the links between parasite traits and the species composition of their host spectra, as well as the links between host traits and the species composition of their parasite assemblages, have seldom been investigated and are, thus, poorly understood. Krasnov et al. (2016) studied trait-based associations between fleas and their small mammalian hosts and found that fleas possessing certain traits exploited hosts possessing certain traits. However, this study did not directly answer the question of whether (a) interspecific similarity in flea traits caused them to exploit similar host species or whether (b) interspecific similarity in host traits caused them to harbour similar flea species, although the results suggested that this could be true. Krasnov et al. (2022) investigated phylogenetic pattern in flea-host interaction networks, i.e. tested for similarity in the sets of associates between phylogenetically closely related species, and reported that the phylogenetic signal for hosts was stronger than that for fleas. In other words, closely related hosts consistently harboured similar flea assemblages, whereas host spectra of closely related fleas were not always similar.

Furthermore, Krasnov et al. (2016) limited their study on flea host trait–based associations to fleas and small mammals of the Palearctic. The patterns of the association between flea or host traits and the species composition of their host spectra or flea assemblages in other biogeographic realms could be different because of substantially different histories of flea-host associations (Medvedev 2005; Whiting et al. 2008a, b; Zhu et al. 2015). Although this appeared not to be the case for phylogenetic signal in flea-mammal interaction networks (Krasnov et al. 2022), the link between trait similarity and similarity in partner composition may not follow the same pattern.

Here, we tested similarity in the species composition of (a) host spectra in functionally similar flea species and (b) flea assemblages in functionally similar host species using data on flea-host interactions from 91 regions of four biogeographic realms (the Afrotropics, the Nearctic, the Neotropics and the Palearctic). Both of these tasks are analogous to the search for a phylogenetic signal in bipartite interaction networks (Ives and Godfray 2006; Peralta 2016), i.e. testing whether closely related species interact with similar partners. Here, we modified this question and considered *closely related species* from a functional rather than a phylogenetic perspective. Consequently, we searched for a *functional signal* in flea-mammal interaction networks using functional dendrograms instead of phylogenetic trees. We also asked whether the probability of detection and the strength of the *functional signal* differ between (a) fleas and hosts and (b) biogeographic realms. Given that phylogenetically closely related species are often similar in their traits (e.g. Krasnov et al. 2011; Surkova et al 2018 for fleas and Olalla-Tárraga et al. 2017; Antoł and Kozłowski 2020) and taking into account the results of Krasnov et al. (2022), we hypothesized that the probability of detection and the strength of the *functional signal* will (a) be stronger for hosts than for fleas and (b) similar between biogeographic realms.

Multiple methods for testing phylogenetic signal in the interaction networks have been proposed (e.g. Cattin et al. 2004; Ives and Godfray 2006; Hadfield et al. 2014; Balbuena et al. 2013; Minoarivelo et al. 2014; Li et al. 2020; Llaberia-Robledillo et al. 2023). Phylogenetic bipartite linear model (PBLM, Ives and Godfray 2006) is one of the most popular of these methods. It has been applied for testing phylogenetic signal in various bipartite networks, both antagonistic and mutualistic (e.g., Martín González et al. 2015; Corro et al. 2021; Michell and Nyman 2021). Recently, Perez-Lamarque et al. (2022) used simulations to test the statistical performances of PBLM and convincingly demonstrated a high (> 30%) rate of false-positive results when using this method. In contrast, an alternative approach of investigating the correlation between a matrix of phylogenetic distances and a matrix of dissimilarity in the species compositions of interacting partners (i.e. the Mantel test) produced much fewer false positives (< 5%), although its statistical power was moderate, especially for small networks. Consequently, Perez-Lamarque et al. (2022) advocated the use of Mantel tests as compared to other methods that either produce too many false positives (e.g. PBLM) or have extremely high computational costs (e.g. Hadfield et al. 2014). Perez-Lamarque et al. (2022) argued that although Mantel tests might not to be able to detect a low phylogenetic signal, the results of these tests can be trusted if the correlations are significant, irrespective of their values. Furthermore, Perez-Lamarque et al. (2022) demonstrated that reliable detection of phylogenetic signal in interaction networks can be achieved if Mantel tests are used with network permutations while keeping the species-specific number of partners constant. In addition, Mantel tests, combined with a Bonferroni correction, as proposed by Perez-Lamarque et al. (2022), can be used to test for phylogenetic signals in separate clades within a large network. Consequently, we applied the approach of Perez-Lamarque et al. (2022) and tested for *functional signal* in flea-mammal networks using Mantel tests.

## Materials and methods

### Data on fleas, hosts and their interactions

Data on fleas, hosts and their interactions were taken from regional surveys of the fleas (Siphonaptera) recorded on small mammals (Macroscelidea, Eulipotyphla, Rodentia and the ochotonid Lagomorpha) used in our earlier study (Krasnov and Shenbrot 2022). Here, we did not consider data from the Australasian and Indomalayan regions because the data on flea functional traits from these realms are largely unavailable. We also did not include in the analyses the ubiquitous fleas (*Xenopsylla cheopis* (Rothschild, 1903), *Xenopsylla brasiliensis* (Baker, 1904), *Nosopsyllus fasciatus* (Bosc, 1800) and *Nosopsyllus londiniensis* (Rothschild, 1903)) associated with synanthropic ubiquitous hosts (*Rattus norvegicus* (Berkenhout, 1769), *Rattus rattus* (L., 1758) and *Mus musculus* L., 1758). We included in the analyses each host species for which at least one flea species was recorded. In total, we used data from 91 regions (see Supplementary Table S1), including 15 Afrotropical regions, 23 Nearctic regions, 17 Neotropical regions and 36 Palearctic regions (see maps and sources of information, Krasnov and Shenbrot 2022).

### Flea and host functional traits and construction of functional dendrograms

By definition, a trait is “any morphological, physiological or phenological feature measurable at the individual level” (Violle et al. 2007). However, information on flea traits according to this definition is unavailable (except for body size and occurrence of combs, see below). Consequently, we used species-specific characteristics/attributes that we further refer to as traits. This is because species-specific attributes are often used as *traits* in many studies of functional diversity (e.g. feeding habit in Pavoine et al. 2016, humidity affinity in Šipoš et al. 2017).

Data on the functional traits of fleas and hosts were taken from Krasnov et al. (2023). We selected flea traits that are presumably associated with their pattern of parasitism, whereas we selected host traits that presumably affect the composition of their flea assemblages (see rationale behind this selection of traits, as well as sources of information on and details of the calculation of some traits in Krasnov 2008 and Krasnov et al. 2016, 2019, 2023). In brief, flea traits were (a) the number of sclerotized ctenidia (no combs, one comb, or two combs); (b) ranked body size (small, medium, or large; based on data for females); (c, d) the number and phylogenetic diversity of a host spectrum across a flea’s entire geographic range; (e) the latitudinal span of geographic range (the difference between the northernmost and the southernmost records) and (f) a preference to spend the most time in a host’s hair or in its burrow/nest or no clear preference. Small mammal traits were (a) average body mass; (b) relative brain mass; (c) geographic range size (log-transformed prior to analyses); (d) location of a nest on, above, or below ground; (d) ground-dwelling, fossorial or arboreal lifestyle, or a combination of lifestyles; (e) diurnal, nocturnal or cathemeral activity; (f) omnivorous, folivorous, granivorous or insectivorous feeding habit, or a combination of feeding habits; (g) occurrence of hibernation or torpor; (h) characteristic population density; (i) average home range size; (j) the dispersal distance (between the birth and the breeding location); (k) characteristic size of a social group and (l) habitat breadth according to level 1 IUCN habitats. All continuous variables for both fleas and mammals were scaled to zero mean and unit variance.

For each realm and either fleas or hosts, we compiled a matrix of species traits (species × traits). Then, from each of these matrices, we constructed a dissimilarity matrix using Gower distance (Gower 1971), which allows the use of different types of variables simultaneously (continuous, categorical, dichotomous, nominal and fuzzy) and is tolerant of missing values. However, using Gower distance often results in a dissimilarity matrix with an unequal contribution of some variables (de Bello et al. 2021). To avoid this, de Bello et al. (2021) modified this procedure and proposed the *gawdis* function, implemented in the *gawdis* package of the R Statistical Environment (R Core Team 2023). This function produces a dissimilarity matrix for multiple variables (traits) with uniform contributions of different variables. We used this function and subsequently built a cluster dendrogram from each dissimilarity matrix using the *hclust* function of the R package *stats* with the option *method* = “*average*”. Then, we transformed each dendrogram into a *functional* (i.e. pseudo-phylogenetic) tree with the *as.phylo* function of the R package *ape* (Paradis and Schliep 2019).

### Data analyses

As mentioned above, we applied the approach of Perez-Lamarque et al. (2022) and tested for *functional signal* in flea mammal-networks using Mantel tests. We controlled for the number of partners per species for both fleas and hosts as recommended by Perez-Lamarque et al. (2022). First, we tested for this signal separately for networks in each realm, using the *phylosignal_network* function implemented in the R package *RPANDA* (Morlon et al. 2016) with options *method* = “*UniFrac_unwejghted*” and *correlation* = “*Pearson*” and ran 10,000 permutations. The input for each test consisted of (a) an interaction binary matrix with flea species in rows and host species in columns, (b) a functional dendrogram for hosts and (c) a functional dendrogram for fleas. Then, we explored *functional signal* in separate clades of each realm-specific functional dendrogram for branches containing at least 15 species using the *phylosignal_sub_network* function of *RPANDA* and the same input and the same options as above. In addition, we carried out the analyses of phylogenetic signal in realm-specific networks using Mantel tests. The aim of this additional analyses was not to repeat the results of Krasnov et al. (2022) but rather to receive some indication about relative effects of phylogenetic closeness versus functional similarity on similarity in partner composition in flea-mammal networks.

In the second stage of the analyses, we searched for *functional signal* in each regional network. Subsequently, we asked whether the number of flea species in a network, the number of host species in a network, a network size (the number of flea species times the number of host species) and/or the proportion of realized interactions affect (a) the probability of detecting a significant *functional signal* in a network and (b) the value of the Mantel correlation (i.e. signal strength) between a matrix of functional distances and a matrix of dissimilarity on partner species composition. The relationships between the abovementioned network properties and (a) the probability of detecting a significant signal or (b) signal strength were analysed using (a) logistic mixed-effects models with the *glmer* function (*family* = *binomial*) and (b) linear mixed-effect models with the *lmer* function, respectively, of the R package *lme4* (Bates et al. 2015) with realm as a random factor. In the latter analyses, only significant Mantel correlation values were included. Initially, we constructed the models with all possible combinations of explanatory variables, as well as intercept-only models. Then, we selected the best model based on the Akaike Information Criterion using the *model.sel* function of the R package *MuMIn* (Bartoń 2023). These analyses were carried out separately for fleas and hosts. The marginal (the proportion of variance explained by the fixed effects) and the conditional (the proportion of variance explained by both fixed and random effects) *R*^*2*^ values (Nakagawa and Schielzeth 2013) for mixed effects models were calculated using the R package *performance* (Lüdecke et al. 2021).

## Results

**Fig. 1:**
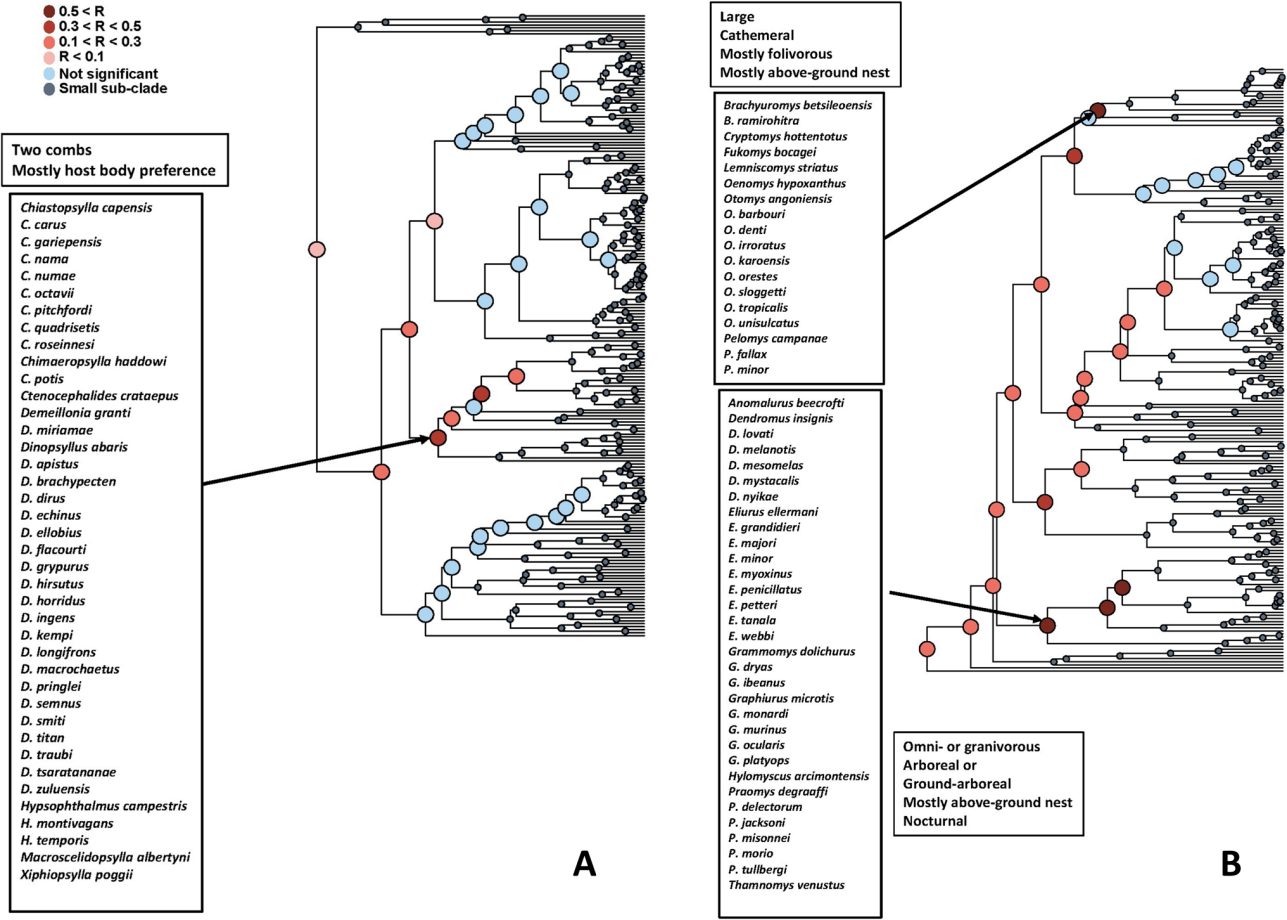
Results of the branch-specific analyses of *functional signals* (see text for explanation) in species interactions for fleas (**A**) and hosts (**B**) in the Afrotropics. The nodes of a functional dendrogram (**A** fleas, **B** hosts) are coloured according to the results of the Mantel test of the correlation (R) between functional dissimilarity and dissimilarity in sets of interacting partners

**Fig. 2:**
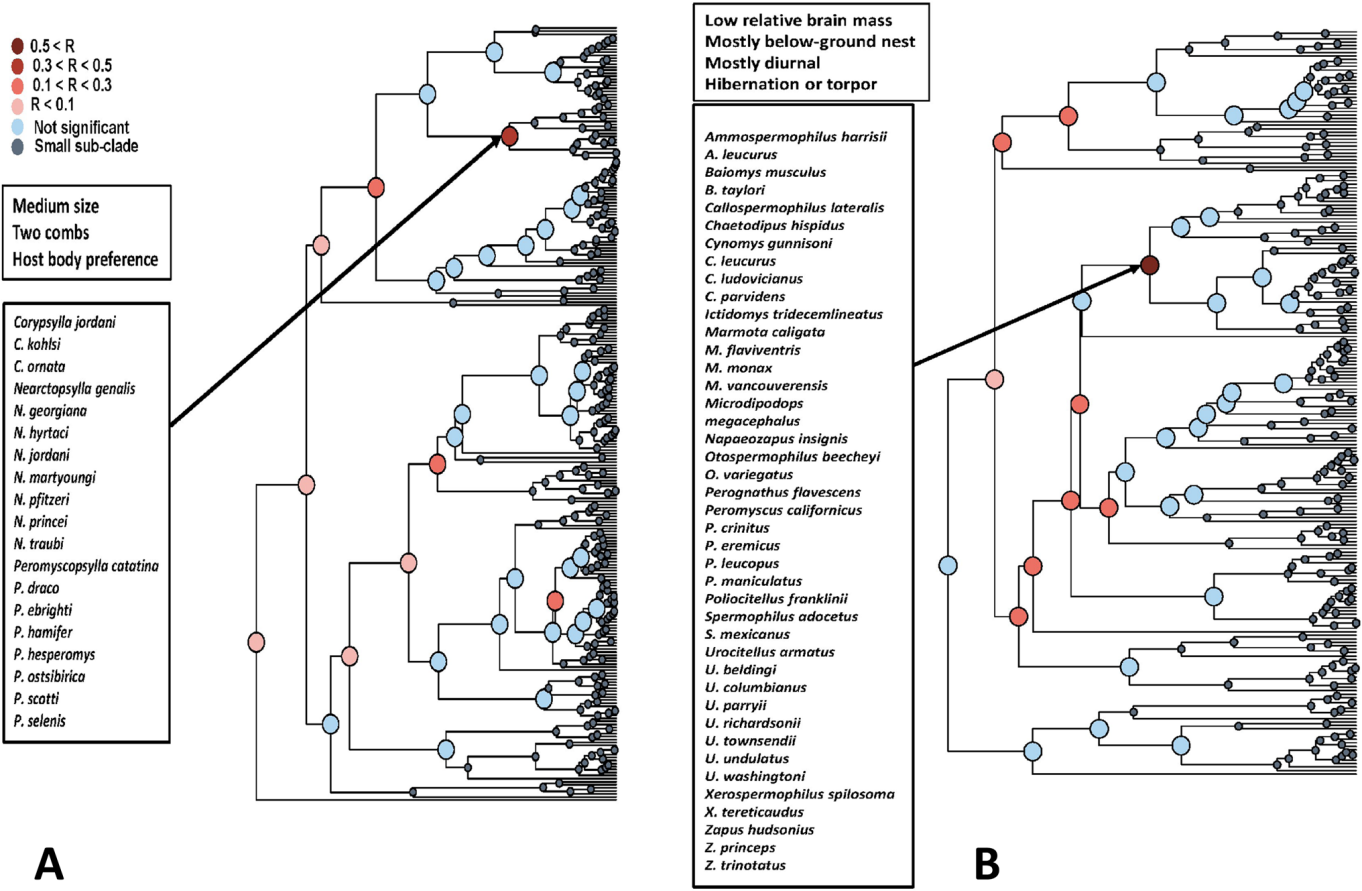
Results of the branch-specific analyses of *functional signals* (see text for explanation) in species interactions for fleas (**A**) and hosts (**B**) in the Nearctic. The nodes of a functional dendrogram (**A** fleas, **B** hosts) are coloured according to the results of the Mantel test of the correlation (R) between functional dissimilarity and dissimilarity in sets of interacting partners

**Fig. 3:**
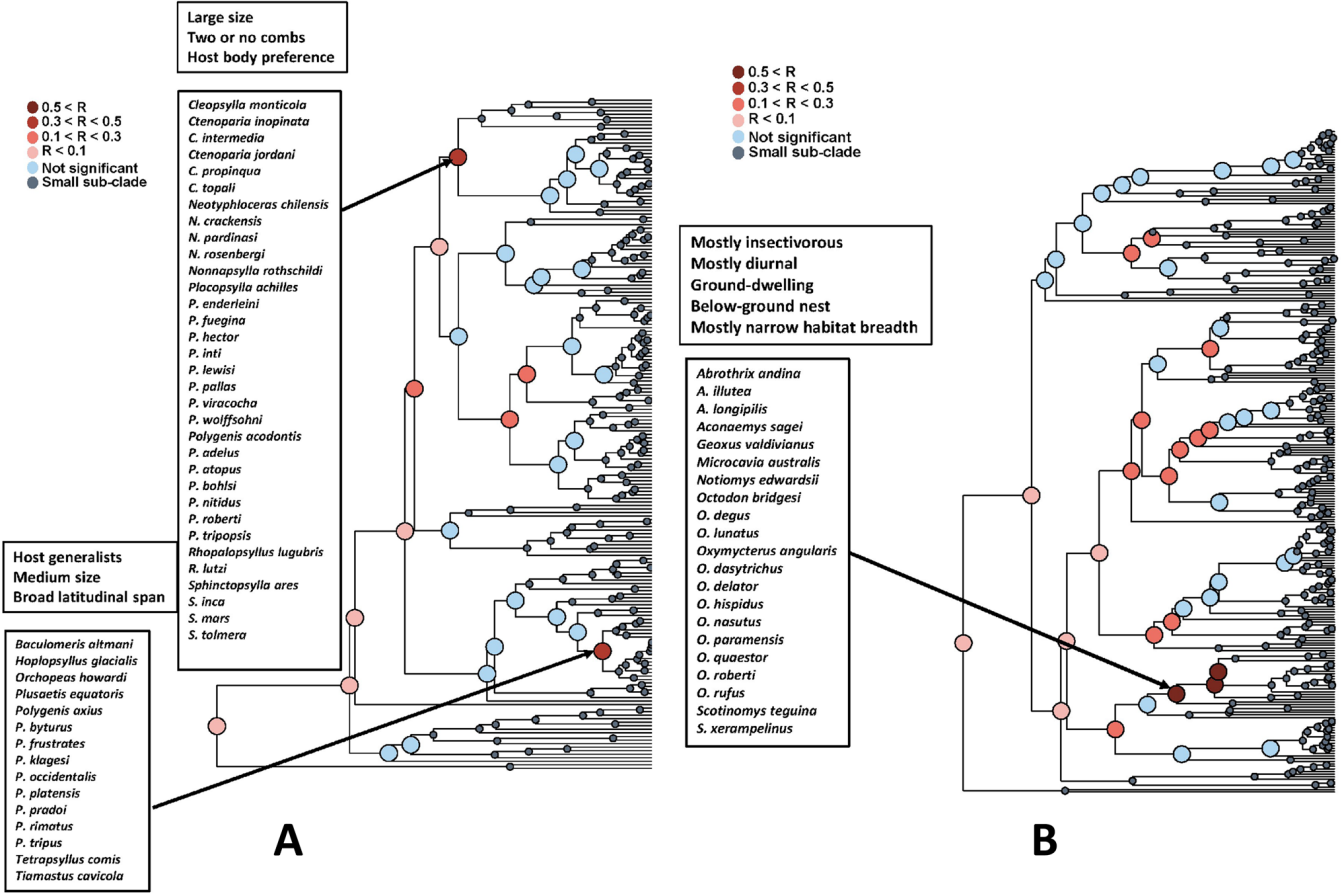
Results of the branch-specific analyses of *functional signals* (see text for explanation) in species interactions for fleas (**A**) and hosts (**B**) in the Neotropics. The nodes of a functional dendrogram (**A** fleas, **B** hosts) are coloured according to the results of the Mantel test of the correlation (R) between functional dissimilarity and dissimilarity in sets of interacting partners

**Fig. 4:**
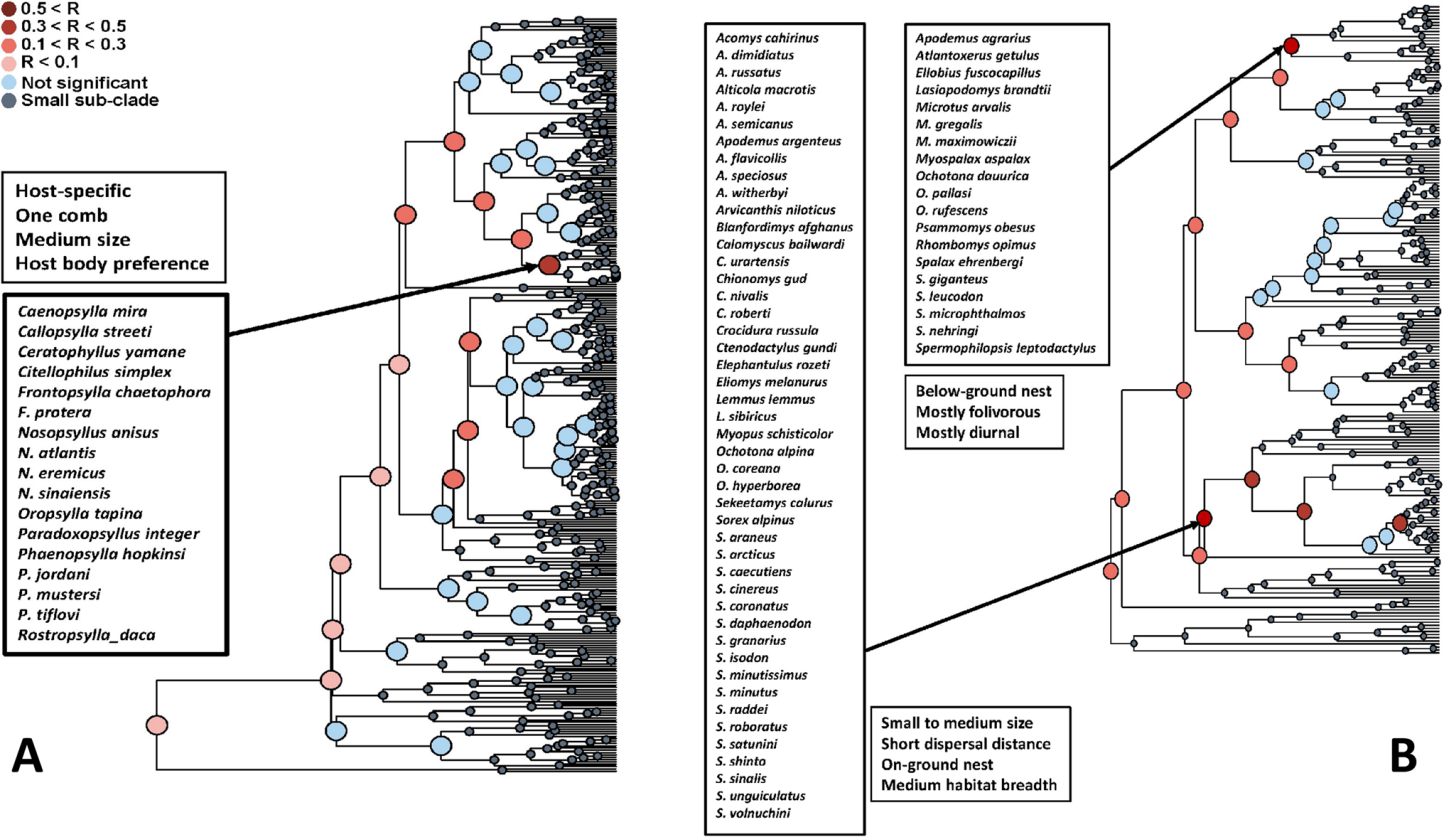
Results of the branch-specific analyses of *functional signals* (see text for explanation) in species interactions for fleas (**A**) and hosts (**B**) in the Palearctic. The nodes of a functional dendrogram (**A** fleas, **B** hosts) are coloured according to the results of the Mantel test of the correlation (R) between functional dissimilarity and dissimilarity in sets of interacting partners

The results of the Mantel test of *functional signal* in realm-specific flea-mammal networks are presented in Table 1. Values of the Mantel correlation were not especially high, but nevertheless significant for both fleas and hosts in all realms. In addition, the correlation values were higher for fleas than for hosts in the Afrotropics and the Palearctic, higher for hosts than for fleas in the Neotropics and equal between fleas and hosts in the Nearctic. The same was true for realm-specific phylogenetic signal except this signal in the Nearctic was higher for fleas than for hosts (Supplementary Table S3). In other words, fleas with similar traits tended to exploit similar hosts, whereas hosts with similar traits tended to harbour similar flea assemblages. However, in each realm, this trend was not characteristic for all fleas and hosts, but rather for some, but not other, clades, i.e. fleas and hosts possessing certain trait sets (Figs. 1, 2, 3 and 4). For example, a similar species composition of host spectra was characteristic for the Afrotropical and the Nearctic fleas preferring to spend most of their time in a host’s hair and having both pronotal and genal combs (Figs. 1 and 2). Analogously, diurnal and folivorous Palearctic hosts possessing below-ground nests harboured similar flea assemblages (Fig. 4). In all realms, functional clades of fleas and hosts that demonstrated a significant *functional signal* were often characterized by a high number of congenerics. For example, the Nearctic clade of medium sized fleas, possessing two combs and having preference for a host’s hair, was composed mainly of *Nearctopsylla* Rothschild, 1915 and *Peromyscopsylla* I.Fox, 1939 (Fig. 2), whereas the Neotropical clade of insectivorous diurnal hosts, with a narrow habitat breadth, was represented mainly by *Abrothrix* Waterhouse, 1837 and *Oxymycterus* Waterhouse, 1837 (Fig. 3).

**Table 1:** Results of testing for *functional signal* in the flea-mammal interaction networks of four biogeographic realms (whether functionally similar species interact with similar partners). *N* number of flea species, number of host species, *M* the Mantel correlation between the functional distances and distances in the composition of either host spectra for fleas or flea assemblages for hosts

Realm	Fleas			Hosts		
	*N*	*M*	*p*	*N*	*M*	*p*
Afrotropics	207	0.15	0.0001	95	0.10	0.0001
Nearctic	257	0.05	0.0001	215	0.05	0.0023
Neotropics	189	0.06	0.0009	258	0.09	0.0001
Palearctic	324	0.16	0.0001	210	0.08	0.0001

**Fig. 5:**
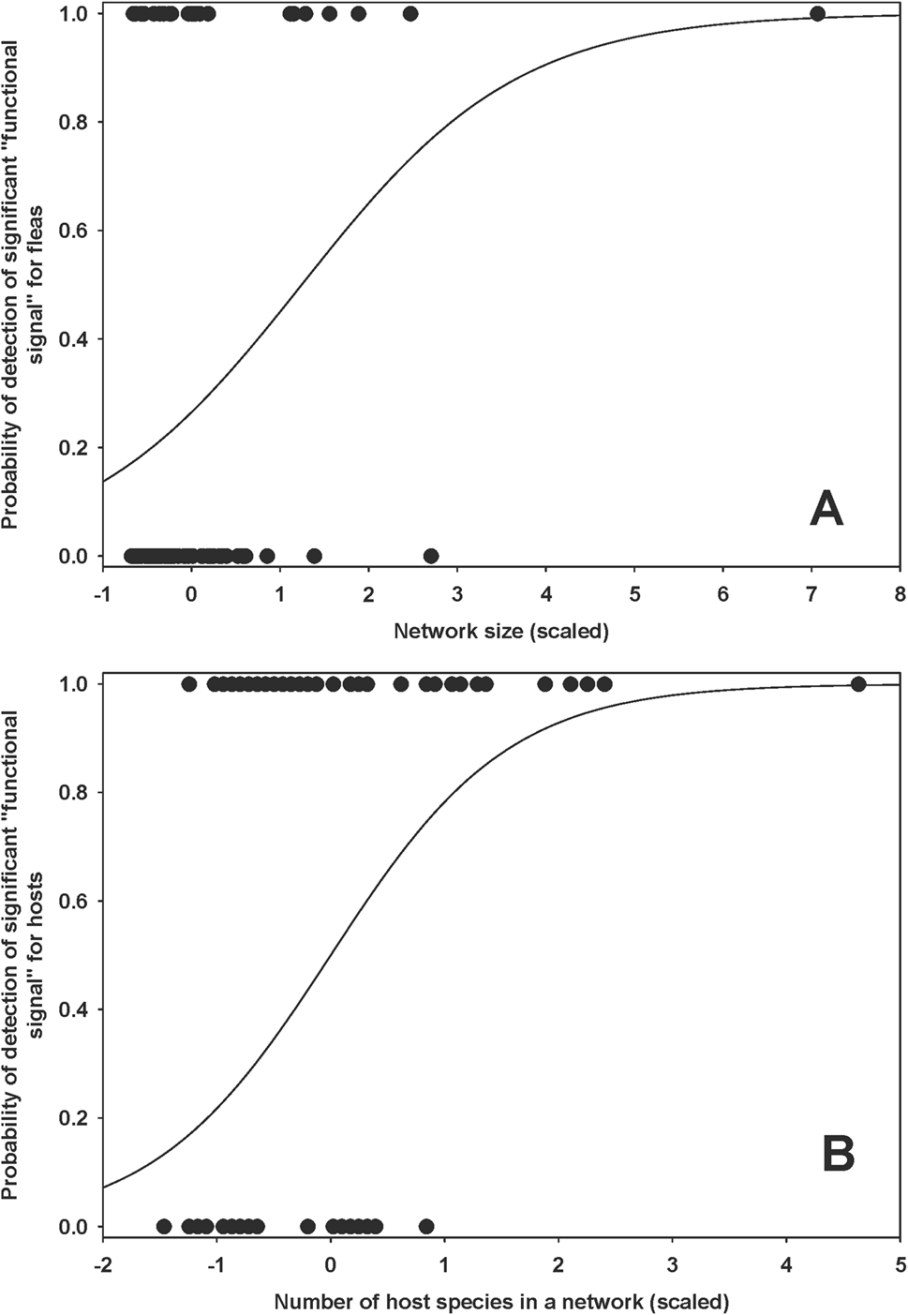
Relationships between the probability to detect a significant *functional signal* (see text for explanation) in a regional flea-mammal network for **A** fleas and network size and **B** hosts and the number of host species

**Fig. 6:**
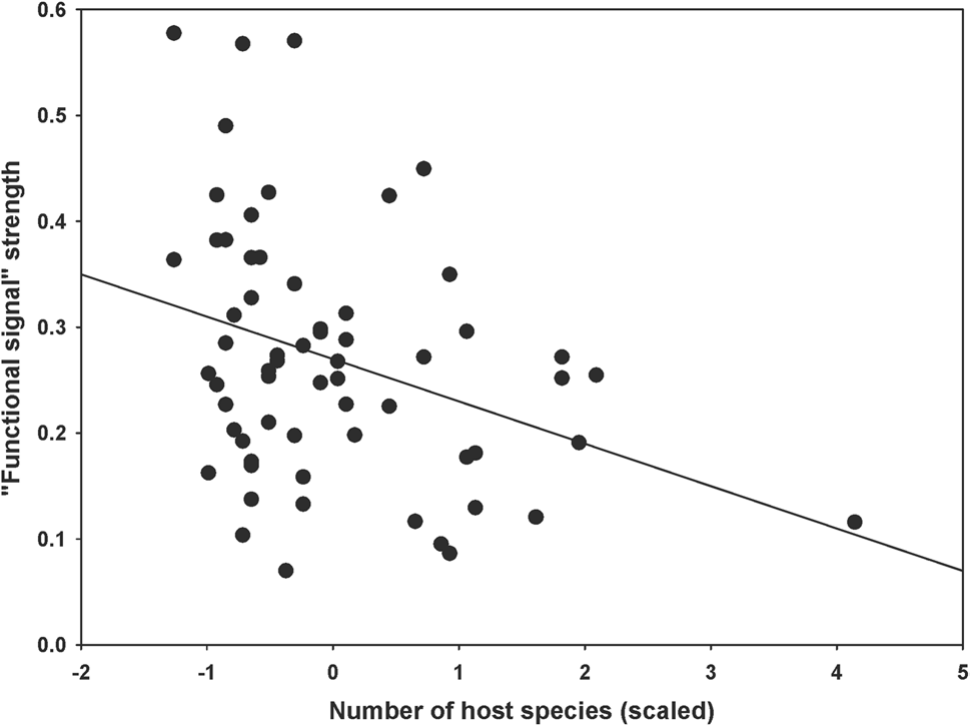
Relationship between the strength of the *functional signal* (value of the Mantel correlation; see text for explanation) in a regional flea-mammal network and the number of host species

*Functional signal* in regional networks was significant in 23 regions for fleas (seven of 15 in the Afrotropics, four of 23 in the Nearctic, two of 17 in the Neotropics and 10 of 36 in the Palearctic) and 63 regions for hosts (nine of 15 in the Afrotropics, 21 of 23 in the Nearctic, eight of 17 in the Neotropics and 25 of 36 in the Palearctic) (Supplementary Table S2). In 18 regions, *functional signal* was significant for both fleas and hosts. For fleas, the probability of detecting a *functional signal* in a regional network increased in larger networks (coefficient = 1.28 ± 0.50, *z*-value = 2.55, *p* = 0.01, marginal *r*^*2*^ = 0.23, conditional *r*^*2*^ = 0.54; Fig. 5a), whereas this probability for hosts increased with an increase in the number of host species (coefficient = 0.81 ± 0.36, *z*-value = 2.25, *p* = 0.02, marginal *r*^*2*^ = 0.16, conditional *r*^*2*^ = 0.22; Fig. 5b). We did not find an association between the strength of the flea *functional signal* and any network property. In contrast, the *functional signal* strength of hosts decreased with an increase in the number of host species (coefficient = 0.04 ± 0.01, *z*-value = 2.63, *p* = 0.03, marginal *r*^*2*^ = 0.10, conditional *r*^*2*^ = 0.20; Fig. 6).

## Discussion

The results of this study partly supported our hypotheses. As we expected, (a) functional similarity in fleas and hosts was associated with the species composition of their partners, and (b) *functional signal* in the regional networks was more frequently found for hosts than for fleas. At the scale of biogeographic realms, these trends were detected in all four networks (albeit with different strength) supporting our expectation. Contrary to our expectations, the frequency of detecting a significant *functional signal* in the regional networks for fleas and hosts differed between realms. For fleas, this frequency correlated positively with the network size, whereas for hosts, it correlated positively with the number of hosts in a network.

### Why is the species composition of partners similar among functionally similar fleas and hosts?

Similarity in the host species composition of fleas similar in their traits and similarity in the flea species composition in hosts similar in their traits suggest a kind of trait matching between fleas and hosts (McQuaid and Britton 2013). In other words, some fleas possess certain sets of traits that allow them to successfully acquire resources from and overcome the defences of their hosts, which may possess certain sets of traits that make them vulnerable to the parasitism of some fleas. It is, however, difficult to identify flea traits that match their host traits and vice versa, although attempts to do this have been undertaken (Traub 1972; Nikitina and Nikolaeva 1979; Krasnov et al. 2016). For example, fleas with two combs (a genal comb and a pronotal comb) and which prefer to spend most of their time in a host’s hair are mainly characteristic of hosts that are able to effectively groom themselves, i.e. hosts with high mobility of their forelimbs. This is because combs allow fleas to anchor themselves in the host hair and resist dislodgement by host grooming (Traub 1972, 1985). This was supported by the findings of Amin and Wagner (1983) that the distance between the tips of flea comb spines correlated positively with the diameter of the host’s hair. From the host perspective, hosts with relatively high brain mass can be attractive to flea species with low abilities of withstanding host immune response because of the negative relationship between investment into *expensive tissue* (e.g. brain) and immunocompetence (Bordes et al. 2011) although this has never been experimentally tested. Fleas preferring to spend most of their time in a host’s burrow are characterised by partial fusion of the metepimeron with the metanotum, lack of genal comb and the elongated labial palpus and mouthparts (Lewis and Spotrono 1984). Some of these characters are observed in *Polygenis* Jordan, 1939 and *Tiamastus* Jordan, 1939 genera that include many species exploiting rodents with fossorial habits that build extensive underground galleries (*Aconaemys* Ameghino, 1891; *Microcavia* Gervais & Ameghino, 1880; *Octodon* Bennett, 1823; *Oxymycterus*) (Hershkovitz 1994). We recognize that these explanations are highly speculative and warrant further investigations. However, it should be noted that the identification of traits complementary between fleas and hosts is hampered by our limited knowledge of the biology and parasitism strategy of the absolute majority of flea species.

In functional clades of fleas and hosts for which a significant *functional signal* was detected, the species composition of host spectra and flea assemblages, respectively, was characterized by a high proportion of congenerics. This suggests that some traits of fleas and hosts that cause them to interact with similar partners are phylogenetically conserved, that is, closely related species (i.e. congenerics) are similar in these traits (see also Krasnov et al. 2022). This was further supported by calculation of phylogenetic (instead of *functional* signal) using the same methodology and the same data as in the main part of current analyses (Supplementary Table S3). For fleas, phylogenetic conservatism has been shown for body size (Surkova et al. 2018), characteristic abundance (Krasnov et al. 2011) and the latitudinal position of geographic range (Krasnov et al. 2018). For mammals, phylogenetic signal was found in various traits, such as body mass, brain mass, dietary diversity, home range and group size (e.g. Olalla-Tárraga et al. 2017; Antoł and Kozłowski 2020). Furthermore, the values of correlation between phylogenetic distances and distances in the composition of partners were higher than those between functional distances and distances in the composition of partners (compare Table 1 and Supplementary Table S3). This suggests relatively stronger effect of phylogenetic closeness than functional similarity in the similarity of partner composition in flea-mammal networks. However, phylogenetic conservatism merely indicates that closely related species are more similar than expected by chance. This does not preclude distantly related species from possessing similar trends. In other words, different phylogenetic lineages may converge in some of their trends. The convergence of ecological traits, for example, a preference to stay mainly in a host’s hair or its burrow for fleas or nest location for hosts, in the independent lineages is associated with natural selection acting on niche-related traits (e.g. Rosenblum et al. 2014). As a result, functional clades of fleas and hosts often consist of groups of congenerics belonging to different genera. For example, the Nearctic functional clade of fleas for which *functional signal* was detected contains species of the genera *Corypsylla* C.Fox, 1908; *Nearctopsylla*; and *Peromyscopsylla* (Fig. 2). All of these species prefer to spend their lives mainly in a host’s hair, have two combs and are of medium size, but the former two belong to the same family, subfamily and tribe (Hystrichopsyllidae: Rhadinopsyllinae; Corypsillini), whereas the latter is a representative of another family (Leptopsyllidae). However, Leptopsyllidae is parafiletic (Whiting et al. 2008a, b), so there is a possibility that pleisomorphic characteristics are being considered as synapomorphies. Similarly, the functional clade of hosts in the Palearctic characterized by small- to medium-sized bodies, short dispersal distance, on-ground nests and medium habitat breadth (Fig. 4) consists of many species of *Sorex* L., 1758 and *Apodemus* Kaup, 1829, which belong to different mammalian orders (Eulipotyphla and Rodentia, respectively). Another (not necessarily alternative) explanation of the high number of congenerics in host spectra of functionally similar fleas (many of which are presumably closely related; see above) is that the majority of flea lineages evolved and diversified on certain host lineages (Traub 1980; Whiting et al. 2008a, b; Zhu et al. 2015). However, hosts from other genera, families or even orders might also be routinely parasitized by functionally similar fleas due to frequent host-switchings, which have been shown to be common events in the history of flea-host associations (Krasnov and Shenbrot 2002; Lu and Wu 2005). In some cases, host-switching followed the dispersal of host lineages (fleas are not capable of dispersing on their own) (Krasnov and Shenbrot 2002; Boyd et al. 2022). In summary, both a flea’s host spectrum and a host’s flea assemblage are formed due to the interplay of evolutionary and ecological processes.

### Difference between biogeographic realms

At the scale of biogeographic realms, the strength of the *functional signal* was higher in the Old World realms than in the New World realms, although this was true for fleas but not for hosts. At the scale of regional networks, the frequency of detecting a significant *functional signal* for fleas was the lowest in the Neotropics and the highest in the Afrotropics, whereas the percentage of regions with a significant *functional signal* for hosts was the lowest in the Neotropics and the highest in the Nearctic. The between-realm difference in the strength of *functional signal* for fleas can be associated with biogeographic patterns of flea evolution. Note that the evolutionary biogeography of fleas obviously depends on that of their hosts but not vice versa. Fleas are thought to have a Gondwanan origin, with South America being the most likely ancestral geographic state of the majority of flea families (Zhu et al. 2015). The long history of fleas in the Neotropics could lead to a kind of homogenization of host spectra in many flea species, resulting in a low (albeit significant) *functional signal* in host species composition. However, the low value of *functional signal* for fleas in the Nearctic cannot be explained in a similar way because the flea fauna of this realm is relatively young, being represented by many species from the most derived families (Leptopsyllidae and Ceratophyllidae) (Medvedev 2005). Possible reasons for the low *functional signal* in the Nearctic could include a relatively (a) low percentage of endemic genera (Medvedev 2005) and (b) a low level of flea specialization (Krasnov et al. 2007). Similarly to the between-realm difference in the *functional signal* strength, the between-realm difference in the frequency of detection of the significant *functional signal* in regional networks for fleas and for hosts could also result from the patterns of fleas’ historical biogeographies, namely a longer and isolated history of flea-host associations in the Neotropics (e.g. Jameson and Fulk 1977) and a shorter history in the remaining realms. Furthermore, although a low level of flea specialization in the Nearctic has led to a low *functional signal* for both fleas and hosts at the scale of the entire realm, as well as to a low frequency of detecting a significant signal for fleas across regions, the frequency of detecting a significant signal for hosts across regions was the highest in this realm. This could be because within a region, each Nearctic host species, on average, interacted with more flea species than in other realms (1.5 versus 0.81–1.22), so the probability of functionally similar hosts to harbour the same fleas could increase.

### Difference between fleas and hosts

The main difference between fleas and hosts regarding *functional signal* was that the significant signal in regional flea-mammal networks for hosts was found more frequently than for fleas. In other words, functionally similar hosts tended to harbour similar flea assemblages more often than functionally similar fleas tended to exploit similar hosts. This is counterintuitive because it is fleas that select their hosts and not vice versa. However, hosts are not submissive victims of parasites, as they defend themselves against parasitism using various tools. The defence efforts of a host might lead to the exclusion of some species from its parasite assemblage, although this has never been empirically proven. In addition, some species drop out from a host’s flea assemblages due to ecological reasons, such as the unsuitability of a host burrow’s microclimate in certain habitats for certain flea species (Krasnov et al. 1997). In other words, a host can directly or indirectly regulate the species composition of its parasite assemblage, at least to some extent. More frequent detection of host than flea *functional signal* in flea-mammal networks could thus arise if these determined-by-the-host processes of flea community assembly are stronger than the determined-by-the-flea processes of host spectrum assembly. Again, we recognize that these suggestions are speculative and require empirical testing.

To the best of our knowledge, this study is the first investigating *functional signal* in parasite-host interaction networks. It opens a perspective for a new line of research aimed at understanding why exactly (a) parasites possessing specific traits select particular hosts and (b) hosts possessing specific traits are exploited by particular parasites. A caveat should be mentioned. The selection of adequate functional traits for answering ecological questions is not always easy. Ideally, these traits must be fitness-related and measurable at the individual level (Llopis-Belenguer et al. 2019). However, information on such traits is not readily obtainable for parasites, in general, and for fleas, in particular. Nevertheless, the use of only a few available traits for fleas in this study allowed us to elucidate general, mostly geographically invariant, trends in host-parasite network ecology.

### Supplementary Information

Below is the link to the electronic supplementary material.

Supplementary file1 (DOCX 39 KB)

## Data Availability

The data on fleas and mammals used in the current study is deposited in Mendeley Data (Krasnov 2023, https\\doi.org\10.17632/dzyvrp7kfh.2). The data on flea traits can be obtained from the corresponding author upon reasonable request.
