# Supplementary material for: Functional similarity affects similarity in partner composition in flea-mammal networks
Source: Parasitol Res. 2024 May 6;123(5):203. doi: 10.1007/s00436-024-08229-7 (PMC11070403; doi:10.1007/s00436-024-08229-7)
Supplement: Supplementary file 1 — Supplementary file1 (DOCX 39 KB) [file 436_2024_8229_MOESM1_ESM.docx]

**Electronic Supplementary Material**

**Functional similarity affects similarity in partner composition in flea-mammal networks**

**Boris R. Krasnov^1*^, Irina S. Khokhlova^2^, M. Fernanda López Berrizbeitia^3^, Sonja Matthee^4^, Juliana P. Sanchez^5^ And Luther Van Der Mescht^6,7^**

^1^Mitrani Department of Desert Ecology, Swiss Institute of Dryland Environmental and Energy Research, Jacob Blaustein Institutes for Desert Research, Ben-Gurion University of the Negev, Sede Boqer Campus, 84990 Midreshet Ben-Gurion, Israel;

^2^French Associates Institute for Agriculture and Biotechnology of Drylands, Jacob Blaustein Institutes for Desert Research, Ben-Gurion University of the Negev, Sede Boqer Campus, 84990 Midreshet Ben-Gurion, Israel;

^3^Programa de Conservación de los Murciélagos de Argentina (PCMA) and Instituto de Investigaciones de Biodiversidad Argentina (PIDBA)-CCT CONICET Noa Sur (Consejo Nacional de Investigaciones Científicas y Técnicas), Facultad de Ciencias Naturales e IML, UNT, and Fundación Miguel Lillo, Miguel Lillo 251, 4000 San Miguel de Tucumán, Argentina;

^4^Stellenbosch University, Private Bag X1, Matieland, 7602, South Africa;

^5^Centro de Investigaciones y Transferencia del Noroeste de la Provincia deBuenos Aires – CITNOBA (CONICET-UNNOBA), Ruta Provincial 32 Km 3.5, 2700 Pergamino, Argentina;

^6^Clinvet International (Pty) Ltd, Uitsig Road, Universitas, Bloemfontein 9338, South Africa;

^7^Department of Zoology and Entomology, University of the Free State, 205 Nelson Mandela Dr, Park West, Bloemfontein 9301, South Africa.

**Supplementary Table S1.** Regions for which data on flea and host species composition were used in the analyses. Name: name of a region, Description: some description of regional components (when needed), Abb: abbreviation of region names used in the cluster analyses in some figures.

| Realm | Name | Description | Abb |
| --- | --- | --- | --- |
| Afrotropics | Angola |  | ang |
|  | Botswana |  | btw |
|  | Congo | Democratic Republic of | cng |
|  | East South Africa | Orange Free State, North West, Gauteng, Mpumalanga, KwaZulu Natal, and Limpopo Provinces | esa |
|  | Ethiopia |  | eth |
|  | Kenya |  | ken |
|  | Madagascar |  | mdg |
|  | Mozambique |  | mzb |
|  | Namibia |  | nmb |
|  | Nigeria |  | ngr |
|  | Coastal South Africa | Eastern Cape and Western Cape Provinces | csa |
|  | Tanzania |  | tnz |
|  | Uganda |  | ugn |
|  | Western South Africa | Northern Cape Province | wsa |
|  | Zimbabwe |  | zmb |
| Nearctic | Alaska | Mainland | als |
|  | British Columbia |  | bcl |
|  | California |  | clf |
|  | Connecticut |  | cnc |
|  | Florida |  | flr |
|  | Georgia |  | grg |
|  | Idaho |  | idh |
|  | Indiana |  | ind |
|  | Maine |  | mai |
|  | Manitoba |  | mnt |
|  | Maryland |  | mrl |
|  | Mexico |  | mxc |
|  | Missouri |  | mss |
|  | Montana |  | mtn |
|  | New Mexico |  | nmx |
|  | Ohio |  | oho |
|  | Ontario |  | ont |
|  | Oregon |  | org |
|  | Tennessee |  | tns |
|  | Texas |  | txs |
|  | Utah |  | uta |
|  | Wisconsin |  | wsc |
|  | West Virginia |  | wvr |
| Neotropics | Argentinian Cuyo | Mendoza, San Juan, and San Luis Provinces | cuy |
|  | Argentinian Mesopotamia | Corrientes, Missiones, and Entre Rios Provinces | msp |
|  | Argentinian North-West | Jujuy, Salta, Catamarca, La Rioja, Santiago del Estero, and Tucuman Provinces | anw |
|  | Argentinian Pampas | Buenos Aires, La Pampa, Santa Fe, Entre Rios, and Cordoba Provinces | apm |
|  | Argentinian Patagonia | Rio Negro, Neuquen, Chubut, Santa Cruz, and Tierra del Fuego Provinces | ptg |
|  | Brazilian Amazonia | Acre, Amazonas, Roraima, Rondonia, Para, and Mato Grosso (north of 15˚S) Provinces | amz |
|  | Brazilian Caatinga | Maranhão, Piauí, Ceara, Paraibo, Alagoas, Sergipe, Bahia, Rio Grande do Norte, Pernambuco, and Minas Gerais (east of 47˚W) Provinces | caa |
|  | Brazilian Cerrado | Goyas, Maranhao, Mato Grosso do Sul, Tocantins, and Minas Gerais (west of 47˚W) Provinces | crd |
|  | Mata Atlantica | Esperito Santo, Rio de Janeiro, Parana, and San Paulo Provinces | mat |
|  | Brazilian Pampas | Santa Catarina and Rio Grande do Sul Provinces | bpm |
|  | Arid Chile | North of 32˚S; Sechura and Atacama Deserts, Puna, Bolivian, and Southern Andean Yungas | ach |
|  | Mediterranean Chile | south of 32˚S; Matorral, Valdivian, and Magellanic Forests | mch |
|  | Colombia |  | clb |
|  | Panama |  | pnm |
|  | Peru |  | per |
|  | Uruguay |  | urg |
|  | Venezuela |  | vnz |
| Palearctic | Adzharia | North Caucasus | adz |
|  | Afghanistan |  | afg |
|  | Akmolinsk region | Kazakhstan, now Nur-Sultan region | akm |
|  | Altai Mountains |  | alt |
|  | Armenia |  | arm |
|  | Azerbaijan |  | azb |
|  | Barguzin | Republic of Buryatia | bar |
|  | Caucasus | The Greater Caucasus | ccs |
|  | Dzungarian Alatau | Kazakhstan | dal |
|  | Eastern Balkhash |  | ebh |
|  | Egypt |  | egp |
|  | Fennoscandia |  | fen |
|  | France |  | fra |
|  | Iran |  | irn |
|  | Italy |  | ita |
|  | Japan |  | jpn |
|  | Korea | Republic of Korea | kor |
|  | Kostroma region | Confluence of the Volga and Kostroma Rivers | kst |
|  | Krasnojarsk region | Eastern Siberia | krj |
|  | Kostanay | Northern Kazakhstan | kos |
|  | Mongolia | Northwestern Khangai | mng |
|  | Morocco |  | mrc |
|  | Moscow region |  | msc |
|  | Moyyunkum Desert | Kazakhstan | moy |
|  | Northern Russian Far East |  | nfe |
|  | Novosibirsk region |  | nov |
|  | Poland |  | pln |
|  | Polar Ural Mountains |  | pur |
|  | Slovakia |  | slo |
|  | Spain |  | spa |
|  | Taimyr |  | tmr |
|  | Central Siberia | Toms and Tumen regions | csi |
|  | Turkey |  | trk |
|  | Tatarstan |  | tts |
|  | Western Sayan Mountains | Southern Siberia | wsy |
|  | Xinjiang | Province of China | xin |

**Supplementary Table S2.** Results of testing for *functional signal* in regional flea-mammal interaction networks from four biogeographic realms (whether functionally similar species interact with similar partners). N: number of species, M: the Mantel correlation between the functional distances and distances in composition of partners. See Supplementary Table S1 for abbreviations of region names.

|  |  | Fleas | | | Hosts | | |
| --- | --- | --- | --- | --- | --- | --- | --- |
| Realm | Region | N | M | *p* | N | M | *p* |
| Afrotropics | ang | 27 | 0.11 | 0.014 | 45 | 0.18 | 0.014 |
|  | btw | 23 | -0.03 | 0.066 | 31 | 0.05 | 0.445 |
|  | cng | 41 | -0.07 | 0.966 | 42 | 0.35 | <0.001 |
|  | esa | 29 | -0.01 | 0.123 | 32 | 0.13 | 0.553 |
|  | eth | 18 | -0.18 | 0.915 | 29 | 0.25 | 0.005 |
|  | ken | 48 | 0.08 | 0.003 | 55 | 0.27 | <0.001 |
|  | mdg | 29 | 0.27 | 0.057 | 39 | 0.27 | <0.001 |
|  | mzb | 21 | -0.13 | 0.636 | 30 | 0.29 | 0.001 |
|  | nmb | 28 | 0.04 | 0.024 | 18 | 0.57 | <0.001 |
|  | ngr | 10 | 0.53 | 0.001 | 15 | 0.16 | 0.067 |
|  | csa | 36 | 0.14 | 0.003 | 27 | 0.24 | 0.055 |
|  | tnz | 37 | 0.07 | 0.189 | 35 | 0.42 | <0.001 |
|  | ugn | 29 | 0.12 | 0.000 | 35 | 0.23 | 0.002 |
|  | wsa | 31 | 0.01 | 0.107 | 30 | 0.10 | 0.090 |
|  | zmb | 18 | 0.03 | 0.028 | 31 | 0.20 | 0.068 |
| Nearctic | als | 27 | 0.01 | 0.521 | 22 | 0.27 | 0.002 |
|  | bcl | 68 | 0.06 | 0.050 | 44 | 0.18 | 0.000 |
|  | clf | 65 | 0.07 | 0.016 | 38 | 0.12 | 0.019 |
|  | cnc | 28 | -0.04 | 0.465 | 19 | 0.41 | <0.001 |
|  | flr | 9 | 0.17 | 0.354 | 10 | 0.36 | 0.033 |
|  | grg | 13 | -0.13 | 0.461 | 16 | 0.38 | 0.001 |
|  | idh | 35 | -0.02 | 0.861 | 19 | 0.37 | 0.001 |
|  | ind | 18 | -0.05 | 0.509 | 22 | 0.27 | 0.001 |
|  | mai | 19 | 0.00 | 0.174 | 19 | 0.14 | 0.034 |
|  | mnt | 35 | -0.11 | 0.980 | 25 | 0.13 | 0.007 |
|  | mrl | 18 | 0.10 | 0.093 | 13 | 0.46 | 0.001 |
|  | mxc | 114 | 0.10 | <0.001 | 89 | 0.12 | 0.004 |
|  | mss | 13 | 0.13 | 0.041 | 18 | 0.19 | 0.027 |
|  | mtn | 41 | -0.01 | 0.620 | 21 | 0.25 | 0.002 |
|  | nmx | 76 | 0.05 | 0.012 | 45 | 0.13 | 0.001 |
|  | oho | 14 | 0.01 | 0.255 | 21 | 0.26 | 0.002 |
|  | ont | 29 | 0.02 | 0.509 | 24 | 0.20 | 0.002 |
|  | org | 76 | 0.03 | 0.077 | 59 | 0.26 | <0.001 |
|  | tns | 18 | -0.10 | 0.867 | 19 | 0.17 | 0.017 |
|  | txs | 11 | 0.06 | 0.275 | 14 | -0.07 | 0.704 |
|  | uta | 63 | 0.04 | 0.313 | 44 | 0.30 | <0.001 |
|  | wsc | 23 | 0.02 | 0.216 | 17 | 0.31 | 0.001 |
|  | wvr | 24 | -0.13 | 0.990 | 19 | 0.17 | 0.041 |
| Neotropics | cuy | 35 | -0.05 | 0.698 | 32 | -0.02 | 0.544 |
|  | msp | 15 | -0.16 | 0.715 | 29 | 0.13 | 0.334 |
|  | anw | 40 | 0.09 | 0.386 | 52 | 0.12 | 0.010 |
|  | apm | 26 | -0.04 | 0.477 | 38 | 0.03 | 0.358 |
|  | ptg | 43 | -0.12 | 0.831 | 32 | 0.19 | 0.078 |
|  | amz | 7 | 0.45 | 0.007 | 17 | 0.03 | 0.348 |
|  | caa | 13 | -0.32 | 0.939 | 27 | 0.25 | 0.001 |
|  | crd | 17 | -0.20 | 0.493 | 41 | 0.10 | 0.048 |
|  | mat | 22 | -0.09 | 0.778 | 57 | 0.19 | <0.001 |
|  | bpm | 12 | -0.38 | 0.967 | 28 | 0.09 | 0.075 |
|  | ach | 27 | 0.05 | 0.130 | 25 | 0.28 | <0.001 |
|  | mch | 55 | -0.04 | 0.691 | 32 | 0.01 | 0.487 |
|  | clb | 29 | -0.01 | 0.576 | 30 | 0.31 | 0.000 |
|  | pnm | 23 | -0.13 | 0.325 | 28 | 0.01 | 0.658 |
|  | per | 39 | 0.01 | 0.113 | 38 | 0.10 | 0.104 |
|  | urg | 13 | -0.10 | 0.095 | 17 | 0.20 | 0.005 |
|  | vnz | 26 | 0.03 | 0.039 | 42 | 0.09 | 0.001 |
| Palearctic | adz | 17 | 0.21 | 0.395 | 10 | 0.55 | 0.062 |
|  | afg | 47 | 0.09 | 0.105 | 30 | 0.23 | 0.026 |
|  | akm | 23 | 0.19 | 0.002 | 18 | 0.10 | 0.011 |
|  | alt | 9 | 0.37 | 0.633 | 23 | 0.07 | 0.002 |
|  | arm | 36 | -0.07 | 0.873 | 16 | 0.29 | 0.018 |
|  | azb | 21 | -0.10 | 0.786 | 11 | 0.32 | 0.106 |
|  | bar | 28 | 0.22 | 0.002 | 15 | 0.38 | 0.012 |
|  | ccs | 76 | 0.06 | 0.012 | 55 | 0.25 | <0.001 |
|  | dal | 22 | 0.20 | 0.130 | 14 | 0.16 | 0.036 |
|  | ebh | 37 | -0.03 | 0.519 | 21 | 0.21 | 0.001 |
|  | egp | 17 | -0.24 | 0.977 | 24 | 0.34 | <0.001 |
|  | fen | 27 | 0.24 | 0.043 | 24 | 0.57 | <0.001 |
|  | fra | 39 | 0.14 | 0.128 | 31 | 0.20 | <0.001 |
|  | irn | 57 | 0.04 | 0.252 | 29 | 0.27 | 0.016 |
|  | ita | 38 | 0.19 | 0.001 | 27 | 0.30 | <0.001 |
|  | jpn | 36 | 0.08 | 0.067 | 18 | 0.26 | 0.054 |
|  | kor | 26 | 0.09 | 0.099 | 16 | 0.17 | 0.328 |
|  | kst | 20 | 0.18 | 0.287 | 15 | 0.25 | 0.003 |
|  | krj | 19 | 0.15 | 0.067 | 10 | 0.58 | 0.016 |
|  | kos | 17 | 0.16 | 0.010 | 16 | 0.23 | 0.011 |
|  | mng | 36 | 0.05 | 0.166 | 18 | 0.21 | 0.074 |
|  | mrc | 19 | -0.08 | 0.448 | 20 | 0.37 | 0.000 |
|  | msc | 14 | 0.21 | 0.101 | 16 | 0.49 | 0.002 |
|  | moy | 28 | -0.01 | 0.092 | 14 | 0.26 | 0.045 |
|  | nfe | 15 | 0.25 | 0.021 | 15 | 0.43 | 0.001 |
|  | nov | 28 | 0.16 | 0.016 | 24 | 0.19 | 0.166 |
|  | pln | 23 | 0.24 | 0.060 | 21 | 0.43 | <0.001 |
|  | pur | 10 | 0.15 | 0.073 | 10 | 0.43 | 0.146 |
|  | slo | 21 | 0.11 | 0.389 | 19 | 0.33 | 0.011 |
|  | spa | 27 | 0.10 | 0.048 | 25 | 0.16 | 0.009 |
|  | tmr | 11 | 0.11 | 0.203 | 12 | 0.18 | 0.177 |
|  | csi | 23 | 0.10 | 0.062 | 27 | 0.30 | 0.002 |
|  | trk | 62 | 0.14 | 0.001 | 39 | 0.45 | <0.001 |
|  | tts | 33 | 0.31 | <0.000 | 28 | 0.28 | 0.080 |
|  | wsy | 28 | 0.10 | 0.021 | 15 | 0.33 | 0.097 |
|  | xin | 13 | -0.11 | 0.804 | 7 | 0.24 | 0.287 |

**Table S3** Results of testing for phylogenetic signal in the flea-mammal interaction networks of four biogeographic realms (whether closely related species interact with similar partners). N: number of flea species, number of host species, M: the Mantel correlation between the phylogenetic distances and distances in the composition of either host spectra for fleas or flea assemblages for hosts

| Realm | Fleas | | | Hosts | | |
| --- | --- | --- | --- | --- | --- | --- |
|  | N | M | *p* | N | M | *p* |
| Afrotropics | 207 | 0.29 | 0.0001 | 95 | 0.20 | 0.0001 |
| Nearctic | 257 | 0.29 | 0.0001 | 215 | 0.12 | 0.0023 |
| Neotropics | 189 | 0.03 | 0.0009 | 258 | 0.10 | 0.0001 |
| Palearctic | 324 | 0.27 | 0.0001 | 210 | 0.11 | 0.0001 |
